# MRI Features of Primary Breast Lymphoma in Patient with Breast Carcinoma for Differential Diagnosis

**DOI:** 10.5334/jbsr.3014

**Published:** 2023-05-02

**Authors:** Subin Lee, Jung Hee Byon, Eun Jung Choi

**Affiliations:** 1 Department of Radiology and Research Institute of Clinical Medicine of Jeonbuk National University-Biomedical Research Institute of Jeonbuk National University Hospital, Jeonbuk National University Medical School, 20 Geonji-ro, Deokjin-gu, Jeonju City, Jeollabuk-Do 54907, South Korea; 2 Department of Radiology, Ulsan University Hospital, University of Ulsan College of Medicine, 25 Deahakbyeongwon-ro, Donggu, Ulsan, 44033, South Korea (now)

**Keywords:** primary breast lymphoma, synchronous, breast carcinoma, DCE-MRI, DWI

## Abstract

**Teaching point:** Homogeneous internal enhancement and washout pattern on DCE-MRI and a low ADC value on DWI in women with breast carcinoma help distinguish primary breast lymphoma from bilateral synchronous breast carcinoma.

## Case History

**Figure 1:**
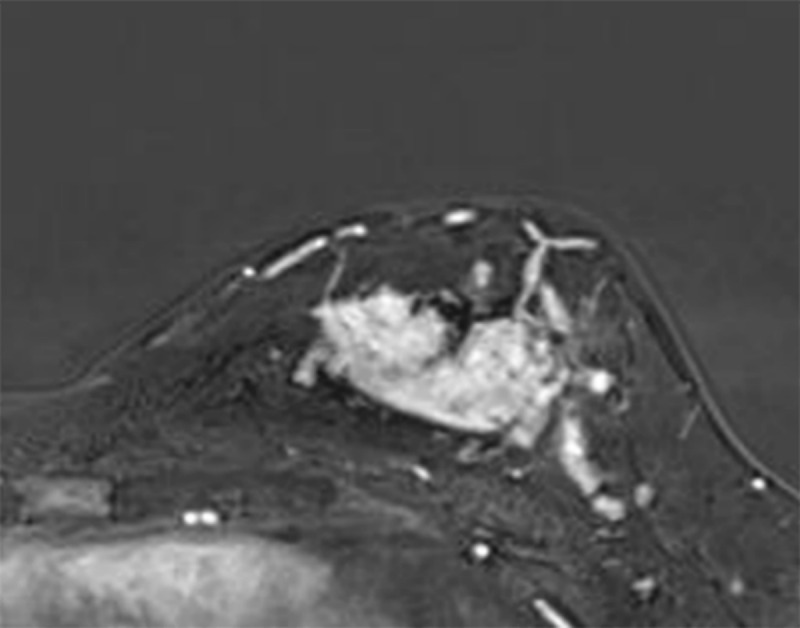
A large mass involving the left breast on DCE-MRI.

A 36-year-old woman was referred to our hospital with a palpable mass in the left breast. Ultrasonography (US) showed a 3.1 × 2.9 cm sized, irregular-shaped, indistinct, and hypoechoic mass. US-guided core biopsy of the left breast mass was performed, and the pathologic result confirmed invasive ductal carcinoma (IDC). Preoperative dynamic contrast-enhanced magnetic resonance imaging (DCE-MRI) showed a 3.8 × 3.2 cm sized, irregular mass with heterogenous enhancement in the IDC (Figure 1), and the mass showed a hyperintense signal on diffusion-weighted imaging (DWI) with an apparent diffusion coefficient (ADC) value of 1.078 × 10^–3^ mm^2^/s.

**Figure 2:**
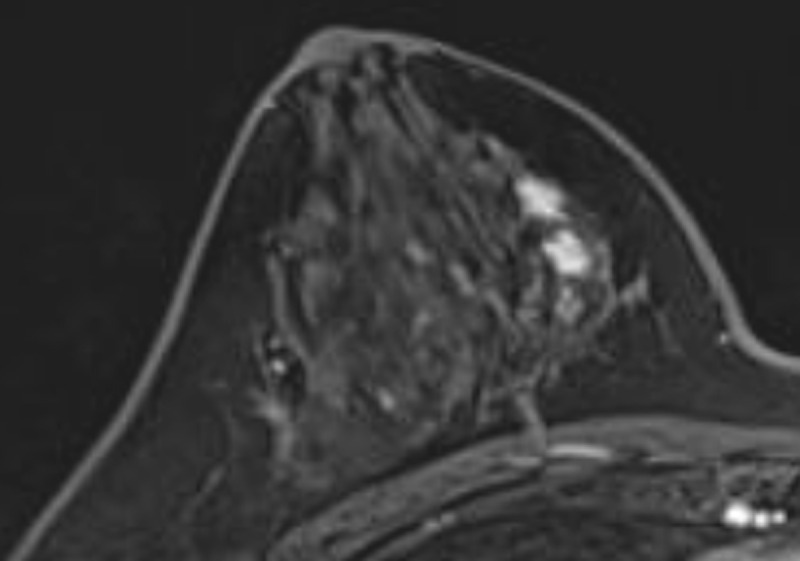
An irregular mass with homogeneous enhancement of the right breast on DCE-MRI.

**Figure 3:**
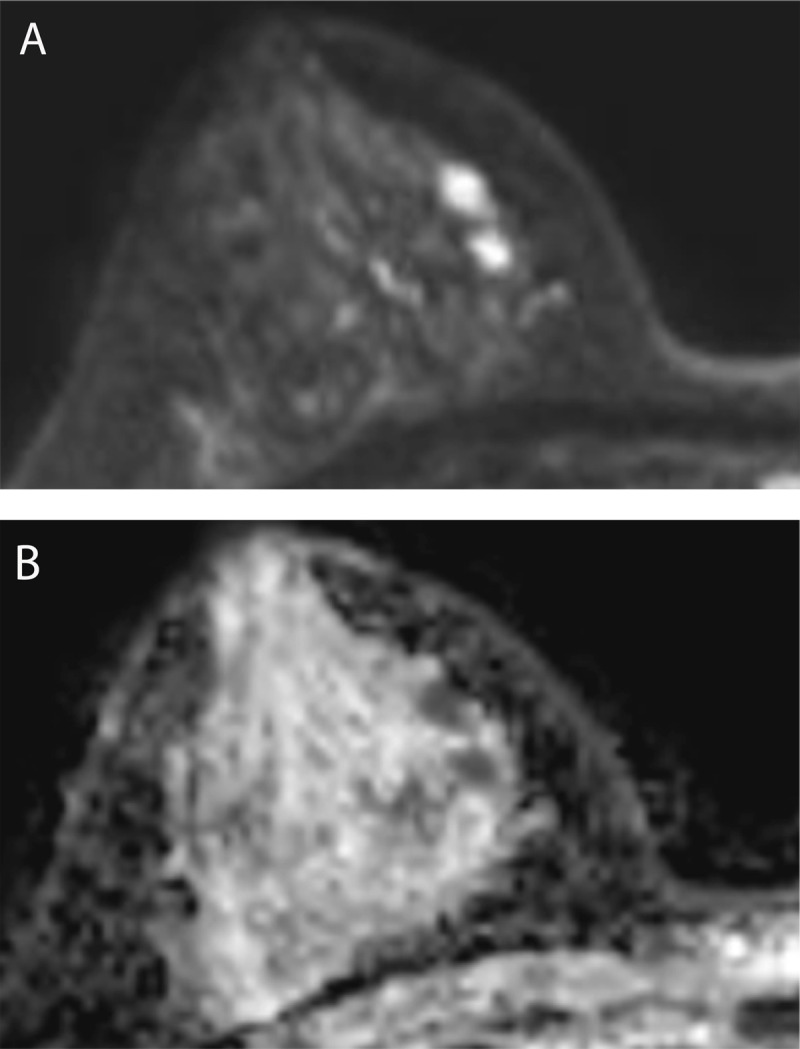
The mass showed a hyperintense signal on DWI with low ADC.

Additionally, a 1.6 × 0.5 cm irregular mass with homogeneous enhancement and a washout pattern on DCE-MRI (Figure 2) and a hyperintense signal on DWI with an ADC value of 0.758 × 10^–3^ mm^2^/s was seen in the right breast (Figure 3).

An approximately 0.8 × 0.4 cm sized, irregular-shaped mass on rescanned US was correlated to the MR lesion of the right breast and showed hypervascularity on the color-Doppler study.

After performing US-guided core biopsy, the pathologic result confirmed mucosa-associated lymphoid tissue (MALT) lymphoma in the right breast.

## Comments

Primary breast lymphoma is an uncommon neoplasm of the breast, occurring in less than 0.6% of primary breast malignancies. Furthermore, the synchronous occurrence of MALT lymphoma and breast carcinoma is extremely rare and has been described in only a few cases in the literature.

Given its rarity, the diagnosis of primary breast lymphoma in a patient with breast carcinoma is considerably challenging. Most primary lymphomas show hyperdense masses without spiculated margins and calcifications on mammography and non-circumscribed hypoechoic masses on ultrasonography, which are frequently considered non-specific [1]. However, DCE-MRI and DWI can be useful in distinguishing lymphoma from breast carcinoma. Internal enhancement of breast lymphomas on DCE-MRI is more commonly homogeneous or mildly heterogeneous, as opposed to breast carcinoma. It is well-known that the mean ADC value is lower in lymphoma than in benign or other malignant tumors such as ductal carcinoma in situ, IDC, and invasive lobular carcinoma because it is a hypercellular tumor. Similarly, the mean ADC value of lymphoma of the right breast was lower than the value for the IDC in the left breast in this case.

In conclusion, homogeneous internal enhancement and washout pattern on DCE-MRI and low ADC values on DWI in women with breast carcinoma can help distinguish primary breast lymphoma from bilateral synchronous breast carcinoma.
